# Zika Virus Infection during Pregnancy and Effects on Early Childhood Development, French Polynesia, 2013–2016

**DOI:** 10.3201/eid2410.172079

**Published:** 2018-10

**Authors:** Lorenzo Subissi, Timothée Dub, Marianne Besnard, Teheipuaura Mariteragi-Helle, Tuxuan Nhan, Delphine Lutringer-Magnin, Philippe Barboza, Céline Gurry, Pauline Brindel, Eric J. Nilles, David Baud, Angela Merianos, Didier Musso, Judith R. Glynn, Gilles Dupuis, Van-Mai Cao-Lormeau, Marine Giard, Henri-Pierre Mallet

**Affiliations:** World Health Organization, Geneva, Switzerland (L. Subissi, P. Barboza, C. Gurry, P. Brindel); Institut Pasteur, Paris, France (T. Dub);; French Polynesia Hospital Center, Pirae, French Polynesia (M. Besnard, D. Lutringer-Magnin);; Institut Louis Malardé, Papeete, French Polynesia (T. Mariteragi-Helle, T. Nhan, D. Musso, V.-M. Cao-Lormeau);; Brigham and Women’s Hospital and Harvard Humanitarian Initiative, Boston, Massachusetts, USA (E.J. Nilles);; World Health Organization, Manila, Philippines (E.J. Nilles, A. Merianos);; University Hospital, Lausanne, Switzerland (D. Baud);; Aix Marseille University, IRD, AP-HM, SSA, VITROME, IHU-Méditerranée Infection, Marseille, France (D. Musso);; London School of Hygiene & Tropical Medicine, London, UK (J.R. Glynn);; University of Québec, Montreal, Québec, Canada (G. Dupuis);; Centre de Liaison sur l’Intervention et la Prévention Psychosociales, Montreal (G. Dupuis);; Bureau de Veille Sanitaire, Direction de la Santé, Papeete (M. Giard, H.-P. Mallet)

**Keywords:** Zika virus infection, Pacific Islands, French Polynesia, congenital Zika syndrome, child health, viruses, early childhood development

## Abstract

Congenital Zika virus syndrome consists of a large spectrum of neurologic abnormalities seen in infants infected with Zika virus in utero. However, little is known about the effects of Zika virus intrauterine infection on the neurocognitive development of children born without birth defects. Using a case-control study design, we investigated the temporal association of a cluster of congenital defects with Zika virus infection. In a nested study, we also assessed the early childhood development of children recruited in the initial study as controls who were born without known birth defects,. We found evidence for an association of congenital defects with both maternal Zika virus seropositivity (time of infection unknown) and symptomatic Zika virus infection during pregnancy. Although the early childhood development assessment found no excess burden of developmental delay associated with maternal Zika virus infection, larger, longer-term studies are needed.

Zika virus is a mosquito-vectored flavivirus first isolated in 1947 in the Zika forest in Uganda (*1*). For the next 60 years, Zika virus was considered to cause sporadic and mild infection in humans (*2*). In 2007, Zika virus emerged in the Western Pacific island of Yap, Federated States of Micronesia (*3*). In 2013, Zika virus emerged in French Polynesia, causing a large outbreak (>30,000 clinical cases estimated during October 2013–April 2014) before spreading rapidly to other Pacific Islands (*4*–*6*). Zika virus emerged in Brazil in 2015 and spread to most of the Americas in 2016 (*7*).

Like some other members of the family *Flaviviridae*, such as West Nile virus and Japanese encephalitis virus, Zika virus is neurotropic (*8*). The link between Zika virus and neurologic disorders such as Guillain-Barré syndrome in adults and microcephaly in newborns is now established (*9*–*15*). Of 84 countries or territories with active autochthonous transmission of Zika virus (as of March 2017), 23 have reported an increase in incidence of Guillain-Barré syndrome, and 31 have reported patients with microcephaly, central nervous system (CNS) malformations, or both potentially associated with Zika virus infection (*16*). After the French Polynesia Zika virus outbreak, health authorities reported an unusual increase in microcephaly and other rare CNS abnormalities of unknown etiology, including corpus callosum or septal agenesis, spina bifida, and brainstem dysfunction (*17*).

Zika virus may be associated with multiple congenital abnormalities (*18*–*20*). The malformations and dysfunctions caused by Zika virus infection during pregnancy are known as congenital Zika syndrome (CZS), but the anatomic, functional, and neurocognitive impairments associated with in utero Zika virus infection have not been precisely defined (*21*). Characterizing the factors contributing to neurocognitive deficits in children born to mothers infected with Zika virus during pregnancy but without overt anatomic malformations, and quantifying the risk of neurocognitive dysfunction, may have major, substantive clinical and public health implications.

We report 2 linked studies conducted in French Polynesia: a retrospective case-control study to determine whether the unusual cluster of CNS congenital defects during and after the Zika virus outbreak in French Polynesia was associated with maternal Zika virus infection, and a cross-sectional study to identify neurocognitive deficits in young children without known birth defects born to mothers who were pregnant during the outbreak. Both studies were powered on the case-control study.

## Methods

### Study Design and Population

#### Case-Control Study

The case-patient definition was any fetus or neonate with a CNS congenital defect of unexplained etiology and a maternal gestational period that overlapped the extended Zika virus circulation period (June 1, 2013–August 31, 2014) by >1 weeks (*17*). Unexplained etiology meant that most commonly suspected etiologies (toxoplasmosis, other, rubella, cytomegalovirus, and herpes [TORCH] infections and genetics) were excluded. We identified cases and controls among fetuses and newborns from the Centre Hospitalier de Polynésie Française in Pirae, French Polynesia, where 60% of all deliveries in this country occur. We identified eligible fetuses from pregnancy terminations using the medical records of the prenatal diagnosis center and eligible newborns from a previously reported case series of congenital cerebral malformations and dysfunctions (*17*) and from the in-hospital discharge records of the neonatology ward and the neonatal intensive care unit. We matched each case-patient to 5 controls by age of the mother at pregnancy (±5 y) and date of conception (date of mother’s last prepregnancy menstrual period ±14 d). We selected the controls randomly from hospital birth records. For logistical reasons, only women residing in Tahiti or Moorea, the 2 most populated and accessible islands, were invited to participate as controls.

#### Cross-Sectional Study

Because a rate of ≈50% Zika virus seropositive mothers was expected in the control group of the case-control study, an additional cross-sectional study was designed to assess whether Zika virus infection in the mother was associated with delayed or abnormal early childhood development (ECD) in the child. Two nurses were trained to conduct anthropometric and neurocognitive testing. ECD was then compared between children with seropositive or seronegative mothers.

### Data Collection

During January–August 2016, mothers of case-patients and controls completed a face-to-face questionnaire on social and economic characteristics, clinical data, and environmental factors, including exposure to chemicals (alcohol, tobacco, drugs, or deltamethrin pesticide spraying) during pregnancy. We retrieved information on seroconversion for *Toxoplasma gondii* and rubella virus during pregnancy from medical files; information on cytomegalovirus (CMV) seroconversion was available for case-patients only. We assessed exposure to deltamethrin by spatiotemporally linking outdoor spraying by the vector control teams with the mother’s residence address during pregnancy. We stratified maternal socioeconomic status as low, medium, or high, adapting from the 4-factor Hollingshead scale (*22*). Gestational age was estimated by each mother’s primary obstetrician, using last menstrual period and first-trimester ultrasound measurements for each mother.

### Laboratory Testing

We detected anti–Zika virus and anti–dengue virus (DENV) neutralizing antibodies in serum from mothers of the case-patients and the controls by using seroneutralization tests, as previously described (*9*,*23*). We incubated serial 2-fold dilutions (from 1:10 to 1:1,280) of each serum sample, previously heat inactivated, for 1 h with strains of Zika virus, DENV-1, DENV-2, DENV-3, or DENV-4. We then inoculated the serum–virus mixtures onto Vero cells and incubated then for 5–7 d. We used ELISA to show the presence of nonneutralized replicative virus in inoculated cells; the reciprocal serum dilution corresponding to the last well showing neutralization activity was the 50% neutralization antibody titer for that serum sample (*23*,*24*). We determined Zika virus infection in mothers by detection of anti–Zika virus neutralizing antibodies (NAb) in serum collected 20–35 months after the expected beginning date of pregnancy. We defined mothers for whom the anti–Zika virus NAb titer was >20 as seropositive and those with a titer <20 as seronegative.

### Assessment of Physical and Developmental Status of Children

We used 4 anthropometric indicators for children’s developmental status: weight for age, height for age, weight for height, and head circumference for age. We calculated Z scores (number of SDs of a value above or below the mean) using WHO Anthro software (*25*). We calculated birth anthropometric indicators (Z scores for weight, height, and head circumference) using INTERGROWTH-21st project software (*26*). We used the French version of the Child Development Assessment Scale, kindly provided by the Centre for Liaison on Intervention and Prevention in the Psychosocial Area, Canada (http://www.ged-cdas.ca), to assess ECD. The Child Development Assessment Scale, which is available in French and is well correlated with the Bayley scale 3rd edition (*27*), consists of questions and observations adapted to children 0–5 years of age and has been validated for use by nonspecialized health or education professionals (Vézina N. Elaboration and validation of the Child Development Assessment Scale, 0–5 years [thesis]. Québec City (QC, Canada): Université du Québec; 2005). The scale is divided into 3 domains: socioemotional; cognitive, including language; and motor. In each domain, children are classified as adequate (no development issues), question (development to be monitored), or problem (specialized pediatric testing required).

### Exposures, Outcomes, and Statistical Analysis

We used 2 measures of exposure: maternal Zika virus seropositivity, a binary variable (yes/no) based on serology results; and reported Zika virus infection, a categorical variable, which used information reported by the mothers (Zika-like illness during or outside pregnancy), associated with serology results, and divided into symptomatic infection during pregnancy; asymptomatic infection, timing unknown; and no infection (seronegative mothers) and symptomatic infection when not pregnant. Zika-like illness corresponded to a clinical diagnosis of Zika virus disease or a recalled infectious episode characterized by rash, fever, or both associated with >2 of the following symptoms: conjunctivitis, arthralgia, myalgia, or limb edema.

#### Case-Control Study

We performed a conditional logistic regression analysis to assess whether CNS congenital defect was associated with the 2 measures of exposure: maternal Zika virus seropositivity and reported Zika virus infection. We conducted a univariate analysis using all identified potential maternal risk factors and confounders (reported chikungunya infection, use of medical or recreational drugs, exposure to deltamethrin during pregnancy, age, socioeconomic status, history of miscarriage and/or termination of pregnancy), as well as parity and history of congenital defects in the family. Variables at p<0.2 in the univariate analysis were further tested by multivariate conditional logistic regression and were retained in the final model if p<0.1. When data sparsity did not allow adjustment for more variables, we retained only those at p<0.05; we grouped maternal middle and high socioeconomic status categories together and compared them with low socioeconomic status. We used a Wilcoxon signed-rank test to compare means between cases and controls and Pearson χ^2^ test to compare categorical variables. We estimated the association between CNS congenital defects and maternal Zika virus infection by matched crude and adjusted odds ratios (ORs) with 95% CIs. We maximized the study power for the case-control study, for which we aimed to include all eligible cases (n = 25), and we chose a control–case ratio of 5:1 to have 98.5% power to detect a bilateral (α = 5%) significant difference, assuming 50% of controls (*28*) and >90% of cases (*9*) were seropositive for Zika virus. The case-control study protocol was approved by the French Polynesia Ethics Committee on February 2, 2016.

#### Cross-Sectional Study

The main outcome was a binary variable derived from the results of the Child Development Assessment Scale (no abnormal development vs. abnormal development). We performed a multivariate logistic regression analysis to assess the associations of ECD with maternal Zika virus seropositivity and reported Zika virus infection. We used the same method as described in the preceding section, with breastfeeding as an additional risk factor/confounder. We performed statistical analyses using Stata version 13.0 (StataCorp LLC, College Station, TX, USA). For the comparison between the French-speaking Canadian children (n = 269) and the sample of our cross-sectional study (n = 107), we had a power of 80% to detect an effect size *d* = 0.32 at α = 0.05. The observed effect size (Cohen *d*) for the cognitive domain is 0.31, and 0.2 for the motor dimension. For the affective dimension, the effect size (ϕ coefficient) is 0.03.

The cross-sectional study on ECD among controls was approved on June 3, 2016. We obtained written informed consent from all mothers.

## Results

### Case-Control Study

**Figure 1:**
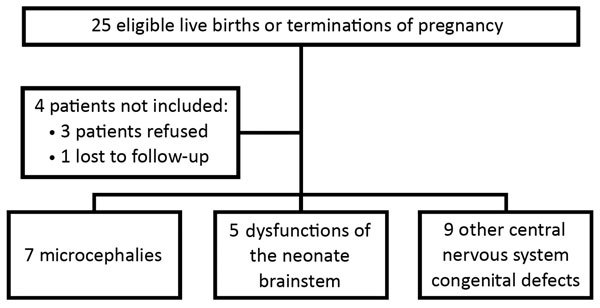
Flowchart for the recruitment of eligible cases for study of Zika virus infection during pregnancy and effects on early childhood development, French Polynesia, 2013–2016.

We identified 26 case-patients; 1 case-patient was later found to have a congenital defect with genetic etiology and thus was no longer eligible and was excluded from the analysis, along with the related 5 controls. The mother of 1 child with microcephaly, already described by Besnard et al. (*17*), as well as the mothers of 2 newborns retrospectively identified with other CNS abnormalities, declined to participate in the study. Another child’s mother was lost to follow-up (Figure 1). Thus, a total of 21 case-patients (84% of those invited) and 102 controls (94%) were enrolled in the case-control study. 

**Figure 2:**
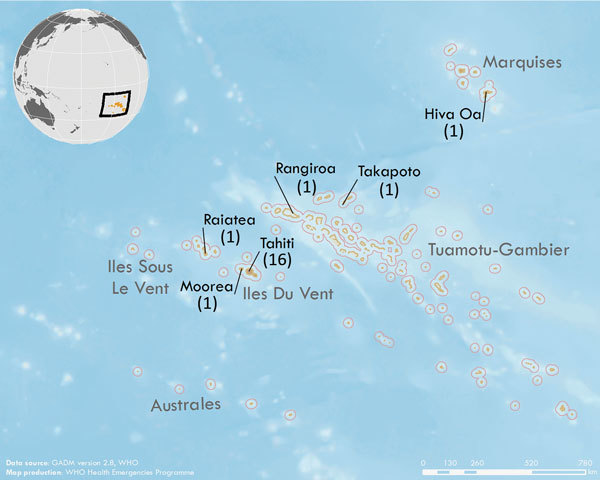
Geographic distribution of eligible cases for study of Zika virus infection during pregnancy and effects on early childhood development, French Polynesia, 2013–2016. Black text indicates islands with >1 case (number of cases from each island is in parentheses); gray text indicates names of archipelagoes. Inset shows the location of French Polynesia in the Pacific Ocean. Data source: GADM version 2.8 (https://gadm.org/download_country_v2.html). Map production: World Health Organization Health Emergencies Programme.

We recruited 5 controls per case-patient for all but 2 case-patients; because of the tight matching and the lack of suitable controls, 1 case-patient had 4 matched controls and 1 case-patient had 3. All controls and 17 case-patients were from Tahiti or Moorea (Figure 2). 

Of the 21 case-patients, 7 had microcephaly, 5 had brainstem dysfunction of the neonate characterized by an inability to suck and swallow, and 9 had other CNS congenital defects (Table 1). Moreover, 6 had ventriculomegaly and 3 had arthrogryposis. Several case-patients had >1 CNS abnormality. For 10 case-patients, pregnancy resulted in termination; of the remaining 11 newborns, 7 were still alive as of July 2017 (Table 1). Seven of the 11 newborns had complete imaging, ophthalmoscopy, evoked otoacoustic emissions, and neuroclinical follow-up. 

**Table 1:** Main and secondary diagnoses for 21 case-patients recruited in case-control study of Zika virus infection during pregnancy and effects on early childhood development, French Polynesia, 2013–2016

Diagnoses	No. (%)	Pregnancy termination	Live births	Child alive,* n = 7
Main diagnoses				
Microcephaly	7 (33)	4	3	1
Brainstem dysfunction of the neonate	5 (24)	0	5	3
Other CNS congenital defects	9 (43)	6	3	3
Septal and/or corpus callosum agenesis	3 (14)	1	2	2
Intraventricular hemorrhage	1 (5)	0	1	1
Cerebral hemorrhage and placental calcifications	1 (5)	1	1	0
Polymalformative syndrome	2 (10)	2	0	0
Complete or sacral spina bifida	2 (10)	2	0	0
Secondary diagnoses				
Ventriculomegaly	6 (29)	4	2	2
Arthrogryposis	3 (14)	3	0	0

*As of July 2017.

Maternal toxoplasma serology was positive for 48% (10/21) of the case-patients and 60% (61/102) of the controls; seroconversion occurred during pregnancy for 1 control. Rubella serology was positive for 81% (17/21) of the case-patients and 94% (96/102) of the controls; no seroconversion occurred in any group. We performed maternal CMV serologic testing for 86% (18/21) of the case-patients: 2 were positive without signs of recent seroconversion, but further testing for CMV in amniotic fluid was negative (*17*). All study participants were seronegative for *Treponema pallidum*. The mother of 1 case-patient was known to be seropositive for HIV before the start of the pregnancy.

The gestational periods of fetuses and infants enrolled in the study started during June 2013–August 2014, and the infants were born during February 2014–May 2015. Median maternal age at conception was 27 years for case-patients and 28 years for controls. Case-patients and controls did not differ significantly in terms of fetal gender, ethnicity, and maternal socioeconomic status. Excluding the cases that ended in termination of pregnancy, gestational age at birth did not differ significantly between case-patients and controls (Table 2).

**Table 2:** Characteristics and comparison of case-patients and controls in study of Zika virus infection during pregnancy and early childhood development, French Polynesia, 2013–2016*

Characteristics	Cases, n = 21	Controls, n = 102	p value†
Mother’s age at pregnancy, median (IQR)	26.8 (22.1–35.7)	27.8 (22.2–33.7)	NA
15–24	8 (38)	37 (36)	NA
25–34	7 (33)	43 (42)	NA
>35	6 (29)	22 (22)	NA
Estimated pregnancy start date, median (IQR)	2013 Dec 11 (2013 Oct 23–2014 May 9)	2013 Dec 8 (2013 Oct 16–2014 May 16)	
January–September 2013	4 (19)	19 (19)	NA
October 2013–December 2014	9 (43)	43 (42)	
January–April 2014	2 (9)	10 (10)	
May–August 2014	6 (29)	30 (29)	
Maternal socioeconomic status			
Low	9 (43)	34 (34)	0.52
Middle	4 (19)	31 (31)	
High	8 (38)	36 (36)	
Child’s birthweight, g	n = 11	n = 100	
<1,500	0	2 (2)	
1,500–2,500	3 (27)	9 (9)	
>2,500	8 (73)	89 (89)	
Child’s sex			
F	10 (48)	42 (41)	0.64
M	11 (52)	60 (59)	
Child’s ethnicity			
Polynesian	14 (74)	61 (71)	0.56
Caucasian	2 (11)	2 (2)	
Mixed/other	3 (16)	23 (27)	
Pregnancy outcome			
Termination of pregnancy	10 (48)	NA	NA
Gestational age at termination of pregnancy, median (IQR)	25.5 (23–29)	NA	NA
Live birth	11 (52)	102 (100)	NA
Gestational age at child’s birth, median (IQR)	39 (36–40)	39 (38–40)	0.23
Term, >37 weeks	8 (73)	85 (83)	
Premature, 27–36 weeks	3 (27)	17 (17)	
Mother’s past infection with dengue viruses			
DENV-1 seropositivity	17 (81)	88 (87)	0.50
DENV-2 seropositivity	12 (57)	50 (50)	0.54
DENV-3 seropositivity	16 (76)	78 (77)	0.99
DENV-4 seropositivity	10 (48)	52 (51)	0.74
Other risk factors/confounders			
Family history of congenital abnormalities	6 (29)	24 (25)	0.84
Drug use during pregnancy‡	9 (45)	34 (33)	0.23
Deltamethrin outdoor spraying during pregnancy	10 (48)	50 (51)	0.73

*Values are no. (%) except as indicated. IQR, interquartile range; NA, not applicable. †Likelihood ratio using conditional logistic regression. ‡Cannabis, cocaine, alcohol, or tobacco.

Maternal Zika virus seroprevalence was 95% among case-patients and 76% among controls (p = 0.07). We classified mothers of 38% of case-patients and 17% of controls as having had symptomatic Zika virus infection during pregnancy and mothers of 57% of case-patients and 60% of controls as having had asymptomatic infection (timing unknown, p = 0.07; Table 3). Of mothers who reported symptomatic Zika virus infection during pregnancy, for case-patients, 88% (7/8) reported it in the first trimester and 12% (1/8) in the second trimester, whereas for controls, 71% (12/17) reported it in the first trimester and 29% (5/17) in the second or third trimester. Compared with those with no evidence of Zika virus infection during pregnancy, the matched crude OR for CNS congenital defects and maternal Zika virus seropositivity was 6.02, and for CNS congenital defects and symptomatic Zika virus infection during pregnancy, the matched crude OR was 6.79. After adjustment for maternal socioeconomic status, these ORs were 7.07 for the first group (95% CI 0.86–58.3; likelihood ratio test p = 0.02), and 7.19 for the second (95% CI 1.39–37.2; likelihood ratio test p = 0.04). Further adjustment for other potential confounders made no difference to the results. Before and after adjustment for confounders, asymptomatic Zika virus infection (timing unknown) was not associated with CNS congenital defects (Table 3).

**Table 3:** Crude and adjusted OR for congenital central nervous system abnormalities and maternal Zika virus infection status, French Polynesia, 2013–2016*

Exposures	Case-patients, no. (%)	Controls, no. (%)	Matched crude OR (95% CI)	Matched adjusted OR† (95% CI)	LRT p value
Zika virus seropositivity	20 (95)	78 (76)	6.02 (0.77–47.1)	7.07 (0.86–58.3)	0.02
Reported Zika virus infection					
No infection during pregnancy‡	1 (5)	24 (24)	1	1	0.04
Asymptomatic (timing unknown)§	12 (57)	61 (60)	2.05 (0.54–7.80)	1.93 (0.47–7.96)	
Symptomatic during pregnancy¶	8 (38)	17 (17)	6.79 (1.36–33.8)	7.19 (1.39–37.2)	

*LRT, likelihood ratio test; NA, not applicable; OR, odds ratio. †Adjusted for maternal socioeconomic status.  ‡Seronegative mothers and seropositive mothers who reported Zika-like illness outside pregnancy. §Seropositive mothers who did not report Zika-like illness during or outside pregnancy. ¶Seropositive mothers who reported Zika-like illness during pregnancy.

### Cross-Sectional Study of Early Childhood Development

More than 1.5 years after the end of the Zika virus outbreak, during June–August 2016, we enrolled 107 children (median age 23 months) in a cross-sectional study and assessed them using the Childhood Development Assessment Scale. Of these children, 44 (41%) were girls, 17 (16%) were born prematurely, and 12 (11%) were classified as having low birthweight (<2,500 g). Except for 1 low birth length baby, anthropometry at birth was within reference range; at the time of evaluation, none of the children was underweight, had low length for age, had low weight for length, or had microcephaly (Technical Appendix Table 1).

We noted evidence for a difference between mean scores of the study participants and the reference population (French-speaking Canadian children) only for the cognitive domain (p = 0.001) (Technical Appendix Table 2). Neurocognitive testing using the Childhood Development Assessment Scale was normal for 93% of the children in the socioemotional domain, 64% in the cognitive domain, and 76% in the motor domain (Technical Appendix Tables 3, 4). We found no evidence for a detrimental effect of maternal Zika virus seropositivity or reported Zika virus infection on ECD in children born without birth defects (Table 4). The weak univariate association of deltamethrin exposure during pregnancy with ECD delays was not significant after adjustment for confounders. Low maternal socioeconomic status (adjusted odds ratio [aOR] 5.28, 95% CI 1.96–14.2) and not breast-feeding (aOR 4.00, 95% CI 1.06–15.1) were associated with abnormal ECD.

**Table 4:** Crude and adjusted odds ratios for maternal Zika virus infection and other risk factors and early childhood development, French Polynesia, 2013–2016*

Risk factors	Early childhood development				
	Adequate in all domains, no. (%)	Question or problem in >1 domain, no. (%)	Adequate in all domains versus question or problem in >1 domain		
			Crude OR (95% CI)	Adjusted OR (95% CI)	LRT p value
Zika virus seropositivity, n = 107					
No	13 (50)	13 (50)	1	1	0.07
Yes	46 (57)	35 (43)	0.76 (0.31–1.84)	0.35 (0.11–1.13)†	
Reported Zika infection, n = 107					
No infection during pregnancy‡	19 (56)	15 (44)	1	1	0.19
Asymptomatic, timing unknown§	30 (54)	26 (46)	1.09 (0.47–2.59)	0.51 (0.16–1.58)†	
Symptomatic during pregnancy¶	10 (59)	7 (41)	0.89 (0.27–2.88)	0.58 (0.14–2.51)†	
Deltamethrin outdoor spraying during pregnancy, n = 104					
No	34 (69)	15 (31)	1	1	0.07
Yes	24 (44)	31 (56)	2.92 (1.30–6.57)	2.69 (0.92–7.84)#	
Maternal socioeconomic status,** n = 106					
Middle and high	47 (66)	24 (34)	1	1	<0.001
Low	11 (31)	24 (69)	4.27 (1.80–10.2)	5.28 (1.96–14.2)††	
Breast-feeding,** n = 106					
Yes, including artificial feeding	52 (57)	39 (43)	1	1	0.03
No	6 (40)	9 (60)	2.00 (0.66–6.09)	4.00 (1.06–15.1)‡‡	

*LRT, likelihood ratio test; OR, odds ratio. †Adjusted for deltamethrin outdoor spraying during pregnancy, maternal socioeconomic status, breastfeeding, and date of pregnancy start (divided into 4 categories based on risk of exposure to Zika virus; see Table 2). ‡Seronegative mothers and seropositive mothers who reported Zika-like illness outside pregnancy. §Seropositive mothers who did not report Zika-like illness during or outside pregnancy. ¶Seropositive mothers who reported Zika-like illness during pregnancy. #Adjusted for ZIKV seropositivity (main exposure), maternal socioeconomic status, breastfeeding, and date of pregnancy start. **No interaction (test for interaction p = 0.26) and no multicolinearity were detected for maternal socioeconomic status and breastfeeding. ††Adjusted for ZIKV seropositivity (main exposure), deltamethrin outdoor spraying during pregnancy, breastfeeding, and date of pregnancy start. ‡‡Adjusted for ZIKV seropositivity (main exposure), deltamethrin outdoor spraying during pregnancy, maternal socioeconomic status, and date of pregnancy start.

## Discussion

We report a case-control study assessing the role of Zika virus infection in CNS malformations, including, but not limited to, microcephaly, in newborns and fetuses whose gestation occurred during the Zika virus outbreak in French Polynesia during 2013–2014. We also report a population-based developmental assessment of children born without birth defects after a Zika virus outbreak.

At the time of the outbreak in French Polynesia, risks associated with reported Zika virus infection were unknown. Four years later, 1 case-control study from Brazil provided evidence that Zika virus infection during pregnancy is associated with microcephaly, and a few cohort studies described Zika virus–associated adverse pregnancy outcomes (*11*,*14*,*18*). Given widespread Zika virus transmission in the Western Hemisphere, clarifying the full spectrum of CZS is a critical public health priority. We conducted our study as part of the World Health Organization (WHO) effort in leading a multicountry coordinated approach to data sharing, surveillance, and research to establish the spectrum of CNS abnormalities attributable to CZS (*29*).

We found evidence that maternal Zika virus seropositivity, with or without reported Zika-like illness during pregnancy, was associated with 7-fold increased odds of congenital CNS defects. Zika virus seroprevalence in the control mothers in the study was 76%, higher than the 49% prevalence detected in the general population of French Polynesia (*28*). Such a difference may exist because the previous Zika virus serosurvey was conducted on a representative subset of the general population, with a median age of 43 years, involving both female and male participants, whereas our study involved only pregnant women, with a median age of 28 years.

Several studies have clearly shown unequal rates of Zika virus infection in men and women, possibly as a consequence of sexual transmission of Zika virus (*30**,**31*). Pregnant women may be more susceptible to Zika virus infection than nonpregnant women of the same age because of the immune tolerance induced by the pregnancy to tolerate paternal antigens (*32*). All the mothers of fetuses or children with microcephaly and other CNS congenital defects were seropositive, whereas 80% (4/5) of the mothers of newborns with brainstem dysfunction were seropositive, compared with 78% (18/23) of their matched controls (matched crude OR 1.05, 95% CI 0.10–11.4). Although no association was found, this finding is inconclusive because the study had little power to perform subgroup analysis by congenital CNS defect. Excluding microcephaly, only multicountry studies or meta-analyses can give a clear answer on the causal link between Zika virus and each reported rare CNS congenital defect (*33*,*34*).

Within the nested cross-sectional study, we assessed control children only on anthropometry and ECD. Both eye and hearing abnormalities have been described in children with CZS; we were unable to test for such abnormalities and cannot infer any conclusion about their burden among children born without diagnosed birth defects in French Polynesia (*35*–*37*).

Our cross-sectional study did not provide evidence that maternal Zika virus seropositivity or symptomatic Zika virus infection during pregnancy were associated with unusual developmental delay in children born without birth defects (Table 4; Technical Appendix Table 4). Known risk factors for developmental delay (low maternal socioeconomic status and lack of breast-feeding) were associated with abnormal childhood development in this study. This result supports the validity of our findings and suggests that if reported Zika virus infection was frequently associated with delayed ECD, we would have likely detected it. However, this study lacked power to detect rare outcomes or minor developmental differences: only 17 control mothers had clear evidence of Zika virus infection with symptoms during pregnancy.

The difference in the cognitive development score in children in French Polynesia compared with children in Canada (Technical Appendix Table 2) is likely to be the result of confounding factors such as socioeconomic status or other population differences; for example, the cognitive scale include items that may be influenced by the cultural context. Furthermore, children in Canada were recruited in Quebec’s public network of kindergartens, which have programs to stimulate children’s development.

The main limitation of our study is the low number of cases (n = 21), which makes the study underpowered to detect a strong association between maternal Zika virus seropositivity and birth defects, as illustrated by the 95% CI crossing the null value. Another limitation is how the exposure was assessed. We used maternal Zika virus seropositivity as a proxy for Zika virus infection during pregnancy, but it is probable that some women were infected with Zika virus outside the gestational period. However, misclassification of mothers, which is likely to be nondifferential assuming they were infected during pregnancy when, in fact, they were infected outside the gestational period, would weaken any association between fetal CNS abnormalities and Zika virus seroconversion. We adjusted for the rapid variations in exposure over a short time by matching controls to cases by date of conception.

Our second measure of exposure, reported Zika virus infection, includes a rough estimate of time of infection (outside or during pregnancy), based on serology data combined with recalled information. This measure may be susceptible to recall bias, because mothers of case-patients are more likely to recall Zika-like illness during pregnancy. However, serology data were compatible with recalled information for both cases and controls (only 1 of 26 mothers who reported Zika-like illness during pregnancy was seronegative). Because of the geographic spread of French Polynesia and the lack of funding, controls could not be residents of any island other than Tahiti or Moorea, which contain >70% of the overall population. We also excluded private hospitals, where 40% of deliveries occur. Therefore, it is likely that socioeconomic status confounded the associations, which is why we adjusted for maternal socioeconomic status in the analysis.

Our study confirms the association between maternal Zika virus infection and CNS congenital defects. Among children with no known congenital defects, we found no evidence that congenital Zika virus infection had a major negative effect on the early stages of childhood development. Because the first large Zika virus outbreak occurred in French Polynesia about 2 years before the Zika outbreaks in Latin America, children exposed to Zika in utero in French Polynesia are now older than those in other countries, but it may still be early to detect subtle developmental delays. Although our data are encouraging, systematic in-depth assessment of childhood development in larger cohorts of exposed children, and at older ages, is needed to detect potential developmental and learning delays.

Technical Appendix Anthropometric scores for case-patients and reference children in study of Zika virus in French Polynesia, 2013–2016.

## References

[1] Dick GWA, Kitchen SF, Haddow AJ. Zika virus. I. Isolations and serological specificity. Trans R Soc Trop Med Hyg. 1952;46:509–20. 10.1016/0035-9203(52)90042-4 12995440

[2] Baud D, Gubler DJ, Schaub B, Lanteri MC, Musso D. An update on Zika virus infection. Lancet. 2017;390:2099–109. 10.1016/S0140-6736(17)31450-2 28647173

[3] Duffy MR, Chen T-H, Hancock WT, Powers AM, Kool JL, Lanciotti RS, et al. Zika virus outbreak on Yap Island, Federated States of Micronesia. N Engl J Med. 2009;360:2536–43. 10.1056/NEJMoa0805715 19516034

[4] Cao-Lormeau V-M, Roche C, Teissier A, Robin E, Berry A-L, Mallet H-P, et al. Zika virus, French polynesia, South pacific, 2013. Emerg Infect Dis. 2014;20:1085–6. 10.3201/eid2011.141380 24856001 PMC4036769

[5] Musso D, Bossin H, Mallet HP, Besnard M, Broult J, Baudouin L, et al. Zika virus in French Polynesia 2013–14: anatomy of a completed outbreak. Lancet Infect Dis. 2018;18:e172–82. 29150310 10.1016/S1473-3099(17)30446-2

[6] Musso D, Cao-Lormeau VM, Gubler DJ. Zika virus: following the path of dengue and chikungunya? Lancet. 2015;386:243–4. 10.1016/S0140-6736(15)61273-9 26194519

[7] Faria NR, Quick J, Claro IM, Thézé J, de Jesus JG, Giovanetti M, et al. Establishment and cryptic transmission of Zika virus in Brazil and the Americas. Nature. 2017;546:406–10. 10.1038/nature22401 28538727 PMC5722632

[8] Tang H, Hammack C, Ogden SC, Wen Z, Qian X, Li Y, et al. Zika virus infects human cortical neural progenitors and attenuates their growth. Cell Stem Cell. 2016;18:587–90. 10.1016/j.stem.2016.02.016 26952870 PMC5299540

[9] Cao-Lormeau V-M, Blake A, Mons S, Lastère S, Roche C, Vanhomwegen J, et al. Guillain-Barré Syndrome outbreak associated with Zika virus infection in French Polynesia: a case-control study. Lancet. 2016;387:1531–9. 10.1016/S0140-6736(16)00562-6 26948433 PMC5444521

[10] Schuler-Faccini L, Ribeiro EM, Feitosa IML, Horovitz DDG, Cavalcanti DP, Pessoa A, et al.; Brazilian Medical Genetics Society–Zika Embryopathy Task Force. Possible association between Zika virus infection and microcephaly—Brazil, 2015. MMWR Morb Mortal Wkly Rep. 2016;65:59–62. 10.15585/mmwr.mm6503e2 26820244

[11] de Araújo TVB, Ximenes RAA, Miranda-Filho DB, Souza WV, Montarroyos UR, de Melo APL, et al.; investigators from the Microcephaly Epidemic Research Group; Brazilian Ministry of Health; Pan American Health Organization; Instituto de Medicina Integral Professor Fernando Figueira; State Health Department of Pernambuco. Association between microcephaly, Zika virus infection, and other risk factors in Brazil: final report of a case-control study. Lancet Infect Dis. 2018;18:328–36. 10.1016/S1473-3099(17)30727-2 29242091 PMC7617036

[12] Cauchemez S, Besnard M, Bompard P, Dub T, Guillemette-Artur P, Eyrolle-Guignot D, et al. Association between Zika virus and microcephaly in French Polynesia, 2013-15: a retrospective study. Lancet. 2016;387:2125–32. 10.1016/S0140-6736(16)00651-6 26993883 PMC4909533

[13] Rasmussen SA, Jamieson DJ, Honein MA, Petersen LR. Zika virus and birth defects—reviewing the evidence for causality. N Engl J Med. 2016;374:1981–7. 10.1056/NEJMsr1604338 27074377

[14] Honein MA, Dawson AL, Petersen EE, Jones AM, Lee EH, Yazdy MM, et al.; US Zika Pregnancy Registry Collaboration. Birth defects among fetuses and infants of US women with evidence of possible Zika virus infection during pregnancy. JAMA. 2017;317:59–68. 10.1001/jama.2016.19006 27960197

[15] Parra B, Lizarazo J, Jiménez-Arango JA, Zea-Vera AF, González-Manrique G, Vargas J, et al. Guillain-Barré syndrome associated with Zika virus infection in Colombia. N Engl J Med. 2016;375:1513–23. 10.1056/NEJMoa1605564 27705091

[16] World Health Organization. Zika situation report. 2017 [cited 2018 Mar 28]. http://www.who.int/emergencies/zika-virus/situation-report/10-march-2017/en/

[17] Besnard M, Eyrolle-Guignot D, Guillemette-Artur P, Lastère S, Bost-Bezeaud F, Marcelis L, et al. Congenital cerebral malformations and dysfunction in fetuses and newborns following the 2013 to 2014 Zika virus epidemic in French Polynesia. Euro Surveill. 2016;21:30181. 10.2807/1560-7917.ES.2016.21.13.30181 27063794

[18] Brasil P, Pereira JP Jr, Moreira ME, Ribeiro Nogueira RM, Damasceno L, Wakimoto M, et al. Zika virus infection in pregnant women in Rio de Janeiro. N Engl J Med. 2016;375:2321–34. 10.1056/NEJMoa1602412 26943629 PMC5323261

[19] Schwartz DA. The origins and emergence of Zika virus, the newest TORCH infection: what’s old is new again. Arch Pathol Lab Med. 2017;141:18–25. 10.5858/arpa.2016-0429-ED 27763793

[20] Moore CA, Staples JE, Dobyns WB, Pessoa A, Ventura CV, Fonseca EB, et al. Characterizing the pattern of anomalies in congenital Zika syndrome for pediatric clinicians. JAMA Pediatr. 2017;171:288–95. 10.1001/jamapediatrics.2016.3982 27812690 PMC5561417

[21] Kapogiannis BG, Chakhtoura N, Hazra R, Spong CY. Bridging knowledge gaps to understand how Zika virus exposure and infection affect child development. JAMA Pediatr. 2017;171:478–85. 10.1001/jamapediatrics.2017.0002 28241263

[22] Hollingshead AB. Four factor index of social status. New Haven (CT, USA): Yale University Department of Psychology; 1975.

[23] Aubry M, Teissier A, Huart M, Merceron S, Vanhomwegen J, Mapotoeke M, et al. Seroprevalence of dengue and chikungunya virus antibodies, French Polynesia, 2014–2015. Emerg Infect Dis. 2018;24:558–61. 10.3201/eid2403.171149 29460745 PMC5823337

[24] Aubry M, Richard V, Green J, Broult J, Musso D. Inactivation of Zika virus in plasma with amotosalen and ultraviolet A illumination. Transfusion. 2016;56:33–40. 10.1111/trf.13271 26283013

[25] de Onis M, Garza C, Victora CG, Onyango AW, Frongillo EA, Martines J. The WHO Multicentre Growth Reference Study: planning, study design, and methodology. Food Nutr Bull. 2004;25(Suppl):S15–26. 10.1177/15648265040251S103 15069916

[26] Cheikh Ismail L, Knight HE, Bhutta Z, Chumlea WC; International Fetal and Newborn Growth Consortium for the 21st Century. Anthropometric protocols for the construction of new international fetal and newborn growth standards: the INTERGROWTH-21st Project. BJOG. 2013;120(Suppl 2):42–7, v. 10.1111/1471-0528.12125 23841804 PMC4084514

[27] Bayley N. Bayley scales of infant and toddler development, 3rd edition. London: Pearson; 2005

[28] Aubry M, Teissier A, Huart M, Merceron S, Vanhomwegen J, Roche C, et al. Zika virus seroprevalence, French Polynesia, 2014–2015. Emerg Infect Dis. 2017;23:669–72. 10.3201/eid2304.161549 28084987 PMC5367400

[29] Costello A, Dua T, Duran P, Gülmezoglu M, Oladapo OT, Perea W, et al. Defining the syndrome associated with congenital Zika virus infection. Bull World Health Organ. 2016;94:406–406A. 10.2471/BLT.16.176990 27274588 PMC4890216

[30] Coelho FC, Durovni B, Saraceni V, Lemos C, Codeco CT, Camargo S, et al. Higher incidence of Zika in adult women than adult men in Rio de Janeiro suggests a significant contribution of sexual transmission from men to women. Int J Infect Dis. 2016;51:128–32. 10.1016/j.ijid.2016.08.023 27664930

[31] Lozier M, Adams L, Febo MF, Torres-Aponte J, Bello-Pagan M, Ryff KR, et al. Incidence of Zika virus disease by age and sex—Puerto Rico, November 1, 2015–October 20, 2016. MMWR Morb Mortal Wkly Rep. 2016;65:1219–23. 10.15585/mmwr.mm6544a4 27832051

[32] King NJC, Teixeira MM, Mahalingam S. Zika virus: mechanisms of infection during pregnancy. Trends Microbiol. 2017;25:701–2. 10.1016/j.tim.2017.05.005 28578821

[33] Baud D, Gérardin P, Merriam A, Alves MP, Musso D, Genton B, et al. Harness shared data in international Zika registry. BMJ. 2016;355:i5319. 10.1136/bmj.i5319 27707728

[34] Panchaud A, Vouga M, Musso D, Baud D. An international registry for women exposed to Zika virus during pregnancy: time for answers. Lancet Infect Dis. 2016;16:995–6. 10.1016/S1473-3099(16)30255-9 27684333

[35] Ventura CV, Maia M, Travassos SB, Martins TT, Patriota F, Nunes ME, et al. Risk factors associated with the ophthalmoscopic findings identified in infants with presumed Zika virus congenital infection. JAMA Ophthalmol. 2016;134:912–8. 10.1001/jamaophthalmol.2016.1784 27228275

[36] Leal MC, Muniz LF, Ferreira TSA, Santos CM, Almeida LC, Van Der Linden V, et al. Hearing loss in infants with microcephaly and evidence of congenital Zika virus infection—Brazil, November 2015–May 2016. MMWR Morb Mortal Wkly Rep. 2016;65:917–9. 10.15585/mmwr.mm6534e3 27585248

[37] de Paula Freitas B, de Oliveira Dias JR, Prazeres J, Sacramento GA, Ko AI, Maia M, et al. Ocular findings in infants with microcephaly associated with presumed Zika virus congenital infection in Salvador, Brazil. JAMA Ophthalmol. 2016;134:529–35. 10.1001/jamaophthalmol.2016.0267 26865554 PMC5444996

