# Supplementary material for: Zika Virus Infection during Pregnancy and Effects on Early Childhood Development, French Polynesia, 2013–2016
Source: Emerg Infect Dis. 2018 Oct;24(10):1850–8. doi: 10.3201/eid2410.172079 (PMC6154169; doi:10.3201/eid2410.172079)
Supplement: Technical Appendix — Anthropometric scores for case-patients and reference children in study of Zika virus in French Polynesia, 2013–2016. [file 17-2079-Techapp-s1.pdf]

Article DOI: https://doi.org/10.3201/eid2410.172079

Zika Virus Infection during Pregnancy and Early Childhood Development, French Polynesia, 2013–2016

Technical Appendix

Technical Appendix Table 1. Anthropometry and comparison between Zika virus seropositive and Zika virus seronegative controls*

Data	All, n = 107 (n,% or median, range)	Zika seropositive, n = 81 (n,% or median, range)	Zika seronegative, n = 26 (n,% or median, range)	p value†
Child corrected age‡ (months)	22.5 (13.4–28.3)	23.2 (13.6–28.3)	19.1 (13.4–25.4)	0.0046
12–14.9	17 (16)	13 (16)	4 (15)	0.009
15–17.9	13 (12)	5 (6)	8 (31)	
18–20.9	12 (11)	10 (12)	2 (8)	
21–23.9	35 (33)	26 (32)	9 (35)	
24–30.9	30 (28)	27 (33)	3 (12)	
Anthropometry at birth	n, % or median, IQR	n, % or median, IQR	n, % or median, IQR	
Weight (kg)	3.254 (2.950–3.682)	NA	NA	
Weight Z score§	1.26 (0.68–2.01)	1.43 (0.81–2.03)	0.99 (0.39–2.01)	0.14
Underweight (Z<u><</u>2 SD)	0 (0)			
Length (cm)	49 (47–50)	NA	NA	
Length Z score	1.01 (0.43–1.64)	1.09 (0.43–1.72)	0.84 (0.00–1.48)	0.19
Stunted (Z<u><</u>2SD)	1 (1)	1 (1)	0 (0)	NA
Head circumference Z score	1.14 (0.44–2.16)	1.16 (0.45–2.28)	0.99 (0.37–1.80)	0.36
Microcephaly (Z<u><</u>2 SD)	0 (0)			
Anthropometry at enrollment	n, % or median, IQR	n, % or median, IQR	n, % or median, IQR	
Weight (kg)	12.0 (10.8–13)	NA	NA	
Weight for age (Z score)	0.46 (–0.17 to 1.07)	0.52 (–0.21 to 1.18)	0.32 (0.01–0.81)	0.61
Underweight (Z<u><</u>2 SD)	0 (0)	NA	NA	
Height/length (cm)	84 (80–87.5)	NA	NA	
Height/length for age (Z score)	–0.05 (–0.89 to 0.69)	–0.06 (–0.89 to 0.62)	0.17 (–0.19 to 0.87)	0.66
Stunted (Z<u><</u>2 SD)	0 (0)			
Weight for height/length (Z score)	0.63 (–0.12 to 1.28)	0.62 (–0.12 to 1.33)	0.66 (–0.12 to 1.14)	0.54
Wasted (Z<-2 SD)	0 (0)			
Head circumference (cm)	48 (47–49)	NA	NA	
Head circumference for age (Z score)	0.57 (0.04–1.33)	0.63 (0.11–1.33)	0.41 (0.04–0.89)	0.38
Microcephaly (Z<u><</u>2SD)	0 (0)			

*IQR, interquartile range; NA, not applicable; SD, standard deviation
† [a]Pearson χ^2 test for proportions and Mann-Whitney test for means
‡ Theoretical age if children were born at term.
§ Z-scores were generated using Intergrowth-21st for anthropometry at birth (*1*) and using the WHO 2005 growth charts (*2*) for anthropometry during the visit. A Z-score is defined as the deviation from the mean value of the gender-specific and age-specific reference populations, divided by the standard deviation for the reference population.

Technical Appendix Table 2. Early childhood development scores for French Polynesian children and reference children aged 12–30 months*

	Reference (n = 269)		Study participants (n = 107)		
ECD domain	Mean score	SD	Mean score	SD	p value†
Socioemotional	90	10	92	10	0.69‡
Cognitive	64	19	57	26	0.001
Motor	64	20	60	20	0.08

*ECD, early childhood development; SD, standard deviation
†*t*-test unless specified.
‡χ^2-test using information on children with suspected “abnormal” development (7/107 among study participants and 21/248 among the reference population). The *t*-test was not done because socioemotional scores were not normally distributed.

Technical Appendix Table 3. Early childhood development and maternal Zika virus infection status, French Polynesia, 2013–2016*

Mothers	1. Socioemotional domain A, n (%)	Q, n (%)	P, n (%)	p value†	2. Cognitive domain A, n (%)	Q, n (%)	P, n (%)	p value	3. Motor domain A, n (%)	Q, n (%)	P, n (%)	p value
All (N = 107)	100 (93)	3 (3)	4 (4)	NA	68 (64)	23 (22)	16 (15)	NA	81 (76)	20 (18)	6 (6)	NA
Zika virus seropositivity												
No	25 (96)	1 (4)	0 (0)	0.63	18 (69)	6 (23)	2 (8)	0.54	19 (73)	6 (23)	1 (4)	0.84
Yes	75 (93)	2 (2)	4 (5)	NA	50 (62)	17 (21)	14 (17)	NA	62 (76)	14 (17)	5 (6)	NA
History of Zika infection												
No infection during pregnancy‡	33 (97)	1 (3)	0 (0)	0.67	24 (71)	8 (24)	2 (6)	0.48	25 (74)	8 (24)	1 (3)	0.26
Asymptomatic (timing unknown)§	51 (91)	2 (4)	3 (5)	NA	33 (59)	12 (21)	11 (20)	NA	44 (79)	10 (18)	2 (4)	NA
Symptomatic during pregnancy¶	16 (94)	0 (0)	1 (6)	NA	11 (65)	3 (18)	3 (18)	NA	12 (71)	2 (12)	3 (18)	NA

*A, Adequate; NA, not applicable; Q, Question; P, Problem
†P values obtained using Fisher's exact test.
‡Seronegative mothers and seropositive mothers who reported Zika-like illness outside pregnancy.
§ Seropositive mothers who did not report Zika-like illness during or outside pregnancy.
¶ Seropositive mothers who reported Zika-like illness during pregnancy.

Technical Appendix Table 4. Crude OR for the association of ECD by domain with Zika virus infection, French Polynesia, 2013–2016*

Category	1. Socioemotional domain† (logistic regression, Adequate vs. Question/Problem) Crude OR (95% CI)	LRT p-value	2. Cognitive domain (ordered logistic regression, Adequate vs. Question vs. Problem) Crude OR (95% CI)	LRT p-value	3. Motor domain (ordered logistic regression, Adequate vs. Question vs. Problem) Crude OR (95% CI)	LRT p value
Zika virus seropositivity	2.00 (0.23–17.4)	0.50	1.51 (0.60–3.81)	0.37	0.87 (0.32–2.36)	0.74
History of Zika virus infection						
Asymptomatic (timing unknown)‡	3.24 (0.36–28.9)	0.50	1.55 (0.60–4.00)	0.66	0.77 (0.27–2.17)	0.63
Symptomatic during pregnancy§	2.06 (0.12–35.1)	NA	1.37 (0.38–4.89)	NA	1.37 (0.35–5.31)	NA

*CI, confidence interval; LRT, likelihood ratio test; OR, odds ratio
†Because of the limited number of observations in the socioemotional domain, Question and Problem categories were grouped together and logistic regression was performed.
‡Seropositive mothers who did not report Zika-like illness during or outside pregnancy.
§Seropositive mothers who reported Zika-like illness during pregnancy.

References

1. Cheikh Ismail L, Knight HE, Bhutta Z, Chumlea WC; International Fetal and Newborn Growth Consortium for the 21st Century. Anthropometric protocols for the construction of new international fetal and newborn growth standards: the INTERGROWTH-21st Project. BJOG. 2013;120(Suppl 2):42–7. PubMed http://dx.doi.org/10.1111/1471-0528.12125

2. de Onis M, Garza C, Victora CG, Onyango AW, Frongillo EA, Martines J. The WHO Multicentre Growth Reference Study: planning, study design, and methodology. Food Nutr Bull. 2004;25(Suppl):S15–26. PubMed http://dx.doi.org/10.1177/15648265040251S103
